# Impact of Maintenance Steroids versus Rapid Steroid Withdrawal in African-American Kidney Transplant Recipients: Comparison of Two Urban Centers

**DOI:** 10.4236/ijcm.2016.73021

**Published:** 2016

**Authors:** W. James Chon, Amishi Desai, Coady Wing, Divya Arwindekar, Ignatius Y. S. Tang, Michelle A. Josephson, Sanjeev Akkina

**Affiliations:** 1 Department of Medicine, Division of Nephrology, University of Chicago Medicine, Chicago, IL, USA; 2 Department of Medicine, Division of Nephrology, Loyola University Medical Center, Maywood, IL, USA; 3 School of Public and Environmental Affairs, Indiana University, Bloomington, IN, USA; 4 Department of Medicine, Division of Nephrology, University of Illinois Hospital & Health Sciences System, Chicago, IL, USA; 5 Department of Medicine, Division of Nephrology, Jesse Brown VA Medical Center, Chicago, IL, USA

**Keywords:** Kidney Transplant, African-Americans, Steroid Maintenance

## Abstract

**Background:**

Rapid steroid withdrawal (RSW) is used increasingly in kidney transplantation but long-term outcomes in African-American (AA) recipients are not well known. We compared 1 and 5 year transplant outcomes in a large cohort of AA patients who were maintained on continued steroid therapy (CST) to those who underwent RSW.

**Methods:**

Post-transplant courses of A as receiving kidney allografts from 2003–2011 at two urban transplant centers in Chicago were followed. Prior to outcome analysis, we used Inverse Probability of Treatment Weights (IPTW) to match the two groups on a set of baseline risk factors. Graft and patient survival, GFR at 1 and 5 years, incidence and type of rejection, incidence of post-transplant diabetes mellitus (PTDM), delayed graft function, CMV and BK viremia were compared.

**Results:**

There were 150 AA recipients in the CST analytic group and 157 in the RSW analytic group. Graft and patient survival was similar between the two groups. Rates of CMV viremia were higher in the RSW compared to the CST analytic group at 1 year. Biopsy-proven acute rejection and PTDM were similar between the RSW and CST groups.

**Conclusions:**

In AA recipients, RSW has similar long-term outcomes to CST.

## 1. Introduction

With the availability of more potent immunosuppressive medications, a number of studies have been published over the last two decades evaluating the role of steroid withdrawal in kidney transplantation. The general consensus is that rapid steroid withdrawal (RSW) when compared to continued steroid therapy (CST) is safe and effective and many centers are moving toward a RSW protocol [1]–[16]. However, the safety and efficacy of RSW remain less well-defined in African American (AA) renal transplant recipients. Studies assessing the efficacy of RSW protocols in AA renal transplant recipients were small, short-term, or not randomized between RSW and CST protocols in AA recipients. Furthermore, studies to date include very few, if any, patients for expanded-criteria donors (ECD), donor after circulatory death (DCD), or recipients with elevated panel reactive antibodies (PRA) at the time of transplantation [17]–[24].

We present data comparing RSW AA recipients to CST AA recipients from two urban centers in Chicago. In the CST group, corticosteroids were tapered to maintenance 5 mg daily dosing by 30 days post-transplantation. In the RSW group, steroids were withdrawn within 5 days post-transplantation. To our knowledge this is the only comparison study between RSW and CST in AA recipients. Further, it represents the largest cohort of AAs and the longest outcome data to date in this population.

## 2. Materials and Methods

### 2.1. Study Population

We retrospectively reviewed data from AA transplant recipients at two Chicago-area academic medical centers from 2003 to 2011. During this period, The University of Chicago followed a continued steroid therapy (CST) protocol, and the University of Illinois employed a rapid steroid withdrawal protocol (RSW). Practice patterns at each center post-transplant are summarized in Table 1. Inclusion criteria for the study were AA transplant recipients at least 18 years of age who received either a deceased donor (including ECD and DCD) or living donor kidney, and were induced with anti-thymocyte globulin. Exclusion criteria included: 1) patients requiring corticosteroids prior to transplantation that were continued after transplant; 2) re-transplants or multi-organ transplants; and 3) positive cross-match and ABO incompatible transplants that required maintenance steroid therapy. The Institutional Review Board at both the University of Chicago and University of Illinois at Chicago approved this study.

### 2.2. Outcomes

Primary end-points included patient, graft, and death-censored graft survival. Secondary end-points included the estimated glomerular filtration rate (eGFR) at 1 and 5 years as determined by the Modification of Diet in Renal Diseases (MDRD) equation, the 1 and 5 year incidence of acute cellular and humoral rejection, and cumulative incidence of post-transplant diabetes mellitus (PTDM) defined as the a fasting glucose >126 mg/dL or random glucose >200 mg/dL requiring the initiation of oral anti-hyperglycemic or insulin based agents after transplant.

### 2.3. Immunosuppression Treatment Protocol

Patients in the CST group were induced with 4 doses of anti-thymocyte globulin (maximum dose 100 mg/day). Either mycophenolate mofetil 1000 mg twice a day or mycophenolate sodium 720 mg twice was used as an anti-proliferative agent. Corticosteroid treatment included intravenous methylprednisolone followed by a taper to maintenance steroid dosing of 5 mg per day at 1 month post-transplant. Patients were maintained on tacrolimus with target 12-hr trough level ranging 6 – 9 ng/ml for the first six months and then 4 – 7 ng/ml thereafter (Table 1).

In the RSW group, patients were induced with 5 doses of 1.5 mg/kg/day anti-thymocyte globulin based on ideal body weight. Mycophenolate mofetil 1000 mg twice a day or mycophenolate sodium 720 mg twice a day was also used as an anti-proliferative agent, and prednisone was tapered quickly from 1 mg/kg/day to 0.25 mg/kg/day and off by post-operative day 6. Patients were maintained on tacrolimus with target trough of 8 – 12 ng/mL in the first 2 months, followed 5 – 10 ng/mL thereafter.

### 2.4. Infection Prophylaxis

The CST group received valganciclovir cytomegalovirus (CMV) prophylaxis for 3 months in the intermediate risk (donor CMV positive/negative and recipient CMV positive) and high risk group (donor CMV positive and recipient CMV negative). All recipients also received trimethoprim/sulfamethoxazole single-strength daily for Pneumocystis prophylaxis in the first 6 months and then three times weekly indefinitely post-transplantation. Fluconazole fungal prophylaxis was provided for 1 month immediately post-transplantation.

The recipients in the RSW group that were at intermediate or high risk for CMV infection received valganciclovir for 6 months and all recipients received trimethoprim/sulfamethoxazole single-strength daily for the first 12 months post-transplant. No fungal prophylaxis is provided after discontinuation of steroids. For both the CST and RSW groups, acyclovir HSV prophylaxis was used for 1 month if recipients were low risk for CMV (donor and recipient CMV negative).

### 2.5. Diagnosis and Management of Rejection

The incidence of rejection was determined by either biopsy-proven rejection or empiric treatment for rejection as described below. The types of rejection were determined using the Banff ’05 criteria when possible. In cases where C4d staining was not done, the Banff ’97 criteria were used. Cases that had both an acute cellular component and antibody-mediated component were categorized as antibody-mediated rejection.

The diagnosis and management of rejection varied slightly between the two groups. In the CST group, all clinically suspected rejections had an ultrasound-guided renal allograft biopsy performed. For Banff 1A or 1B acute cellular rejection (ACR), patients were treated with methylprednisolone 500 mg daily for 3 days followed by a quick taper. Banff 2A or greater ACR were treated with anti-thymocyte globulin. For the RSW group, treatment for clinically suspected rejection without biopsy or a borderline/Banff 1A or 1B ACR included methylprednisolone 500 mg daily for 3 days without an oral taper. Patients with Banff 2A or greater ACR were treated with anti-thymocyte globulin, dosed according to ideal body weight. As per protocol, subjects with first time rejections in the RSW group were not started on oral steroid therapy.

### 2.6. Statistical Analysis

To assess the baseline differences in patients from the two centers, we examined the difference in means and standardized difference in means (Cohen’s D statistic) for a set of demographic and clinical characteristics determined prior to the transplant. The two samples were out of balance with respect several important risk factors. To reduce bias from these baseline differences, we estimated propensity scores using a logistic regression of treatment group membership (RSW/CST) on a list of donor, recipient, and transplant factors including recipient age, gender, body mass index, history of diabetes, pre-transplant dialysis, time on dialysis, primary renal disease, PRA ≥ 30%, Hepatitis C; donor age, gender, race, body mass index (BMI), type; HLA matches, CMV risk, and transplant era. Next, we used inverse probability of treatment weights (IPTW) to assign a weight to each member of the CST group (control). To avoid instability from very large weights, the weights in the CST group were normalized and individuals were trimmed from the sample if their weight represented more than 5% of the sum of the weights. To maintain symmetry, we also excluded individuals in the RSW group (treatment) if they had propensity scores greater than the minimum propensity score among the individuals who were trimmed from the control group. We examined several candidate specifications for the propensity score model and selected the specification that achieved the most balanced sample. Categorical and continuous outcomes were estimated using IPTW for proportions and means, respectively, and with 95% confidence intervals.

We used Kaplan-Meier analysis with IPTW to compare graft and patient survival between the two groups. A Cox proportional hazards model using IPTW (unadjusted model) was used to estimate the effect of the maintenance regimen on survival with adjustments for recipient (model 1), donor, and transplant covariates (model 2). The addition of covariates into the Cox proportional model with IPTW provides a doubly robust estimation that increases the chances of an accurate estimation of the outcome [25].

## 3. Results

### 3.1. Patient Characteristics

From 2003 to 2011, 194 patients from the CST group and 212 from the RSW group were initially included in the study. Table 2 shows that, prior to IPTW and trimming, the CST group had more deceased donors, especially DCD, more time on dialysis, fewer black donors, and higher proportion of individuals with a PRA > 30%. After applying IPTW and trimming, there were 150 individuals in the analytic CST group and 157 in the analytic RSW group.

Prior to outcome analysis, a basic question is whether the analytic sample is sufficiently balanced for sound causal inference. There is no universal standard to apply, of course. However, from a clinical perspective, the analytic sample appears well balanced with respect to key risk factors. In addition, the standardized mean difference in covariates (Cohen’s D) is well below 0.25 standard deviations for each of the baseline covariates so that the remaining imbalances is not “too large” from the perspective of one common rule of thumb in the statistical matching literature [26] [27]. In fact, after IPTW and trimming, most baseline covariates have Cohen’s D statistics less than 0.10 standard deviations and the most imbalanced variable has a Cohen’s D of 0.16 standard deviations.

### 3.2. Survival

**Figure 1:**
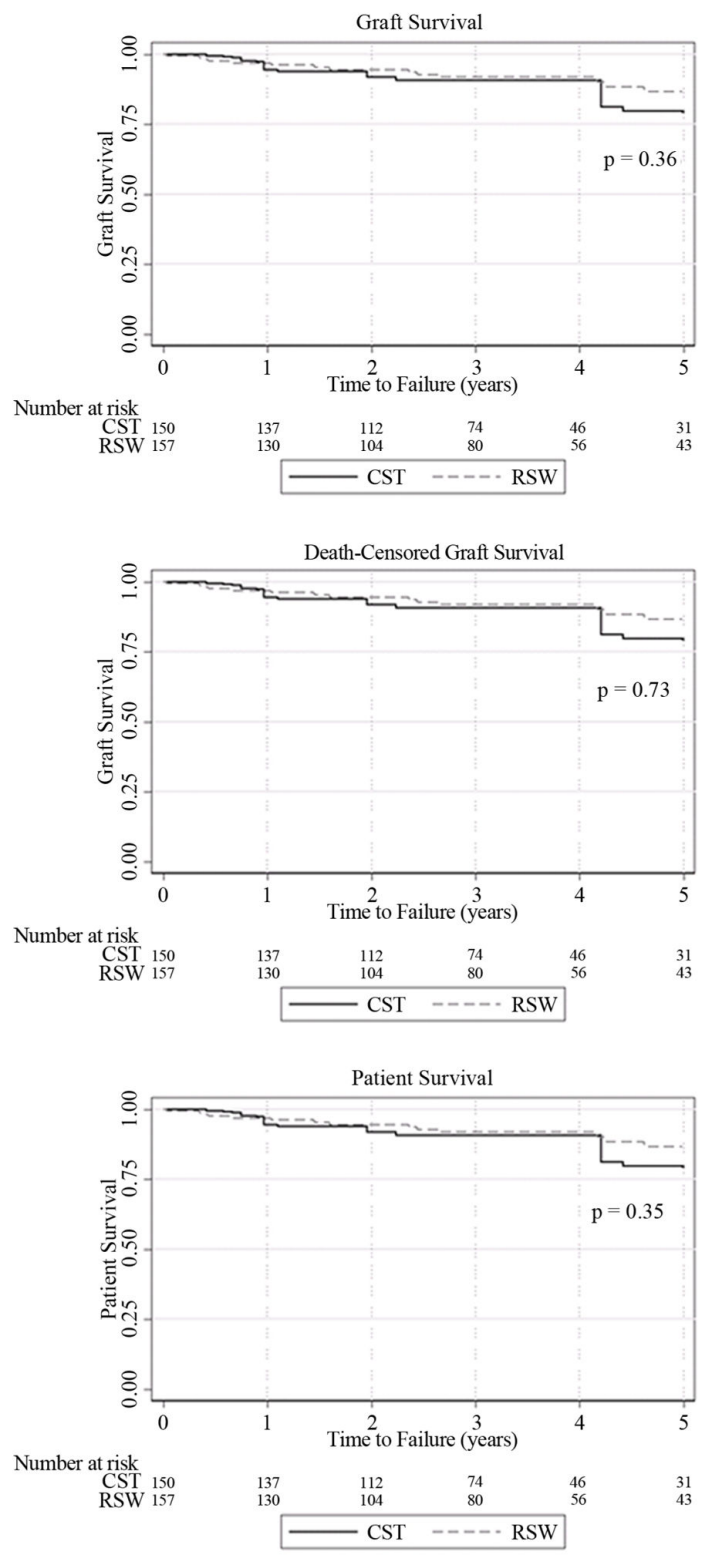
Graft and patient survival all transplants. Kaplan-Meier survival curves adjusted for baseline differences using inverse probability of treatment weights. CST-continued steroid therapy group, RSW-rapid steroid withdrawal group. Log-rank p-value is reported for each analysis.

Graft, death-censored graft, and patient survival were assessed at 1, 3, and 5 years after transplant. In the CST analytic group, there were 25 graft failures and 24 deaths during the follow up period while the RSW analytic group had 24 graft failures and 18 deaths (Table 3). The graft survival between the two centers was similar during the follow up period. The 1, 3, and 5 year graft survival was 93%, 79%, and 59% in the CST analytic group and 94%, 84%, and 72% in the RSW analytic group (Figure 1). Death-censored graft survival showed similar results with 1, 3, and 5 year survival at 99%, 87%, and 74% in the CST analytic group and 97%, 92%, and 83% in the RSW analytic group. Patient survival was 94%, 91%, and 79% at 1, 3, and 5 years for the CST analytic group and 97%, 92%, and 87% in the RSW analytic group. The Cox proportional hazard models for graft and patient survival did not show any significant difference in survival between the two analytic groups (Table 4).

### 3.3. Causes of Graft Failure and Death

The causes of graft failure and patient death with a functioning graft are shown in Table 3. Overall, the causes of graft failure were similar between the two groups with interstitial fibrosis/tubular atrophy being the most common followed by acute rejection. The causes of death were difficult to determine for a number of cases, particularly in the RSW group. However, it appeared that the most common causes were either cardiovascular, infectious, or due to malignancy.

### 3.4. Renal Function

The eGFR at 1 and 5 years after transplant are shown in Table 5. Overall, the eGFR were not statistically different between the two groups at 1 and 5 years after transplant. However, when we stratified subjects into type of kidney transplant, recipients of deceased donor kidneys in the CST analytic group had a higher eGFR compared to deceased donors in the RSW analytic group at 1 year (64.1 vs 52.7 mL/min/1.73 m^2^, p = 0.047) that was no longer significant in those that survived out to 5 years (CST 55.3 vs RSW 51.3 mL/min/1.73 m^2^, p = 0.59). Living donor kidney transplants were not different at either 1 or 5 years after transplant.

### 3.5. Acute Rejection

The incidence of rejection and types of rejection are shown in Table 5. Compared to the CST analytic group, the RSW analytic group had a higher rate of rejection at 1 (32% vs 10%, p < 0.001) and 5 years (44% vs 15%, p < 0.001) compared to the CST analytic group. When limited to biopsy-proven rejections in the first year, the rates were similar (18% vs 10%, p = 0.10). The types of biopsy-proven rejection varied by group with more grade 1 and borderline rejections in the RSW analytic group while grade 2 rejections were more common in the CST analytic group.

### 3.6. Other Complications

The incidence of delayed graft function was much higher among the CST analytic group compared to the RSW analytic group (32% vs 10%, p < 0.001) (Table 5). The incidence of post-transplant diabetes mellitus was similar between the two groups (25% vs 22%, p = 0.66). In regards to viral infections, CMV viremia was more commonly found in the RSW analytic group compared to the CST analytic group (24% vs 11%, p = 0.036) while BK viremia was similar between two groups (18% vs 10%, p = 0.15).

### 3.7. Return to Corticosteroid Therapy

Among the RSW group, 20 of the 157 individuals were started on prednisone after their transplant (Table 6). The most common cause of starting prednisone was rejection (35%). The next most common cause was leukopenia at 25% while BK nephropathy and GI intolerance to mycophenolate mofetil or mycophenolate sodium were each 10%. Of the 20 individuals, 13 (65%) were started in the first year of transplant.

## 4. Discussion

The use of RSW has become more common practice in the management of renal allografts to prevent the side effects of prolonged corticosteroid use. Of patients transplanted in 2011, nearly 40% of recipients were discharged off steroid maintenance with nearly 30% remaining steroid-free at 1 year post-discharge [28] [29]. This study represents the first comparative study of RSW versus CST in AA recipients. A similar number of recipients were included from both centers within similar age ranges. However, there were notable differences in the types of transplants performed at each center. The CST group included more patients with deceased donor kidneys, donor after circulatory death kidneys, increased HLA mismatches and a higher proportion of PRA > 30% recipients as well as recipients receiving renal replacement therapy at the time of transplant when compared to the RSW group. To reduce these biases, we used propensity score weighting with inverse probability of treatment weights to adjust for these differences and then incorporated covariates into a regression model for a doubly robust estimation.

There were few statistically significant differences in allograft function or rejection outcomes within the first year of transplantation (Table 5). Allograft function appeared superior within the CST group at 1 year but the difference was marginal in those that survived out to 5 years. Graft, death-censored graft survival, and patient survival did not appear to be significantly affected by the use of maintenance steroids. The RSW group had a higher rate of CMV viremia while the CST group had a higher rate of delayed graft function. The combined empiric and biopsy-proven acute rejection rate was higher in the RSW group although the incidence of biopsy-proven acute rejection was similar between the two cohorts. Among the biopsy-proven acute rejections, borderline or grade 1 rejections were more likely to be seen in the RSW group while the CST group had more grade 2 rejections, similar to what has been previously reported in other comparison studies.

The practice of using an RSW protocol has improved corticosteroid related complications in renal transplant recipients [8] [9] [21] [30]–[33]. Surprisingly, the incidence of PTDM development remained equal between centers. One would have expected increased post-transplant diabetes in the CSW group but this was not seen in our retrospective analysis. This may be explained, in part, by the higher percentage of patients with Hepatitis C and higher BMI in the RSW group, two established risk factors in the development of PTDM [34]. Further, when compared to the larger steroid doses of the past, a daily dose of 5 mg of prednisone may not significantly contribute to the development of insulin resistance post-transplant [35]. Glycemic control was not assessed in this study but others have shown worse control in those maintained on steroids compared to early withdrawal [32] [33] [36].

Few studies have specifically addressed the clinical outcomes in AA kidney transplant recipients with regards to RSW [17]–[23]. A small, single-center study comparing 56 AA vs. 56 non-AA recipients on varying immunosuppression regimens showed acceptable rejection rates and patient-/graft-survival when prospectively followed up to 3 years [22]. Additionally, long-term outcomes have been published when comparing protocol biopsies between AA and non-AA recipients on RSW protocols [19] [20]. Both studies indicated favorable graft and patient survival outcomes in low PRA AA recipients when compared to non-AA recipients. More recently, a study of 634 recipients of which 27% of patients were AA, showed AA race to be associated with increased rejection and graft loss. Unlike previous studies, 55% of total recipients received deceased donor kidneys of which 46% of the 55% transplanted were from expanded criteria donors [17]. The risk of recurrent disease was similar between the two groups but this subgroup was small. In a few reported studies, the risk of recurrence of glomerulonephritis was similar between steroid maintenance and steroid withdrawal groups [37] [38] except in IgA nephropathy where the risk of recurrence was higher in steroid withdrawal groups [39] [40].

While this study includes one of the largest cohorts of African-Americans, the study does have some limitations. First, the study was conducted as a retrospective chart review at two centers where differences in practice may have affected the results. The major difference is type of kidney donors used between the groups, specifically the higher percentage of deceased donors in the CST group compared to the percentage of living donors within the RSW group. To minimize these differences, we used propensity score weighting to match individuals between the two groups and removed matched samples where a few individuals in the control group represent a disproportionately high number in the treatment group. We then adjusted for the covariates along with propensity score weighting to give a doubly robust estimation to further minimize the biases. Other variations include CMV management post-transplant and goal trough levels of tacrolimus being different between groups. Interestingly, CMV viremia was seen more in the RSW with the longer valganciclovir prophylaxis. And while we were unable to gather the data on trough levels in either group, the lower target levels of tacrolimus in the CST group may explain why eGFR was higher then in this group. Finally, the high number of empirically treated rejections in the RSW group likely underestimates the biopsy-proven rejection rate since other studies of steroid withdrawal have shown higher rejection rates. Despite these significant differences in practice between these two centers where these differences could bias the outcomes in favor of the CST protocol, we still found no difference in graft and patient survival. However, more prospective, long-term, controlled studies are needed to confirm these findings before recommending the routine use of RSW protocols in African-American kidney transplant recipients.

## 5. Conclusion

We conclude that a RSW regimen is comparable in graft and patient survival to a CST regimen in AA recipients.

## Figures and Tables

**Table 1:** Practice differences between the CST and RSW centers.

	CST	RSW
Induction Therapy	Anti-thymocyte globulin 1.5 mg/kg × 4 doses (maximum 100 mg/ day)	Anti-thymocyte globulin 1.5 mg/kg (ideal body weight) × 5 doses (no maximum dose)
	Methylprednisolone	Methylprednisolone
Maintenance immunosuppression	Tacrolimus (trough level) 0 – 6 months – 6 – 9 ng/mL >6 months – 4 – 7 ng/mL	Tacrolimus (trough level) 0 – 2 months – 8 – 12 ng/mL >2 months – 5 – 10 ng/mL
	Mycophenolate mofetil 1000 mg bid or mycophenolate sodium 720 mg bid	Mycophenolate mofetil 1000 mg bid or mycophenolate sodium 720 mg bid
	Prednisone taper to 5 mg daily by 1 month	Prednisone tapered off by day 6
Infection Prophylaxis	CMV Valganciclovir for 3 months Acyclovir × 1 month for low-risk	CMV Valganciclovir for 6 months Acyclovir × 1 month for low-risk
	Pneumocystis TMP/SMX single strength daily × 6 months TMP/SMX single strength three times weekly indefinitely	Pneumocystis TMP/SMX single strength daily × 12 months
	Fungal Oral Fluconazole × 1 month	Fungal Oral nystatin × 5 days
Diagnosis and Management of Rejection	Biopsy-proven only	Empiric or biopsy-proven
	Banff 1A/1B Methylprednisolone 500 mg daily × 3 days Oral taper afterwards	Banff 1A/1B/Empiric Methylprednisolone 500 mg daily × 3 days
	Banff 2A/2B/3 Anti-thymocyte globulin	Banff 2A/2B/3 Anti-thymocyte globulin

**Table 2:** Patient demographics between continued steroid therapy (CST) and rapid steroid withdrawal (RSW) centers.

	Unmatched Cohort			Matched Cohort		

	CST (n = 194)	RSW (n = 212)	Cohen’s D	CST (n = 150^a^)	RSW (n = 157)	Cohen’s D
Age at Transplant (Years)	49.5 (13.4)	51.0 (12.6)	0.12	51.7 (15.2)	51.4 (13.1)	−0.02
Male Recipient	58%	65%	0.14	60%	62%	0.04
Body Mass Index (kg/m^2^)	29.4 (6.3)	30.5 (7.1)	0.16	29.7 (7.1)	30.2 (7.1)	0.07
Deceased Donor	89%	49%	−0.81	69%	62%	−0.14
Extended Criteria Donor	15%	15%	−0.01	18%	17%	−0.03
Donation after Cardiac Death	25%	5%	−0.90	7%	7%	0.00
Diabetes	31%	41%	0.20	32%	39%	0.13
Cause of ESRD						
Diabetes	25%	31%	0.13	26%	30%	0.09
Hypertension	51%	50%	−0.02	50%	50%	0.01
FSGS	9%	6%	−0.13	5%	6%	0.05
Other	15%	14%	−0.05	19%	13%	−0.16
Hepatitis C	4%	12%	0.26	8%	8%	0.02
Dialysis Pre-Transplant	96%	85%	−0.32	93%	94%	0.04
Time on Dialysis (Years)	4.7 (3.2)	3.4 (3.4)	−0.38	4.2 (3.2)	3.9 (3.3)	−0.08
Donor Age (Years)	41.2 (15.4)	38.3 (14.9)	−0.19	39.5 (15.8)	39.6 (15.6)	0.01
Black Donor	26%	60%	0.70	47%	54%	0.14
Male Donor	62%	53%	−0.18	55%	59%	0.07
Donor BMI	28.3 (7.1)	28.9 (6.5)	0.09	28.0 (7.2)	28.1 (6.4)	0.02
HLA Matches						
0 – 2	64%	53%	−0.21	56%	56%	0.00
3 – 4	28%	37%	0.17	35%	36%	0.02
5 – 6	8%	10%	0.07	9%	8%	−0.03
PRA > 30%	22%	8%	−0.55	11%	10%	−0.06
CMV Risk						
Low	11%	6%	−0.22	9%	8%	−0.04
Intermediate	79%	84%	0.14	83%	84%	0.03
High	10%	10%	0.00	8%	8%	−0.01
Transplant Era						
2003–2005	26%	25%	−0.04	27%	25%	−0.05
2006–2008	37%	30%	−0.16	27%	32%	0.11
2009–2012	37%	46%	0.18	46%	43%	−0.06

Reported as either mean (standard deviation) or proportions.

a Weighted total, rounded to the nearest whole number.

**Table 3:** Graft failure and deaths between the continued steroid therapy (CST) and rapid steroid withdrawal (RSW) group.

	CST (n = 150^a^)		RSW (n = 157)		

	n^a^	Incidence (95% CI)	n	Incidence (95% CI)	p-Value
Graft Failure	25	17% (9% – 28%)	24	15% (10% – 22%)	0.82
Cause of Graft Failure					0.64
Acute Rejection	3	14% (3% – 42%)	7	29% (14% – 51%)	
Primary	3	14% (3% – 42%)	1	4% (0% – 26%)	
Infectious	0	0%	1	4% (0% – 26%)	
Recurrence	5	21% (4% – 60%)	2	8% (2% – 29%)	
Interstitial Fibrosis/Tubular Atrophy	9	37% (14% – 68%)	8	33% (17% – 55%)	
BK Nephropathy	3	13% (2% – 51%)	4	17% (6% – 38%)	
Other	0	1% (0% – 11%)	1	4% (0% – 26%)	
Deaths with a Functioning Graft	24	16% (8% – 27%)	18	11% (7% – 18%)	0.40
Cause of Death					0.51
Cardiovascular	8	34% (10% – 70%)	3	17% (5% – 42%)	
Infectious	4	18% (5% – 50%)	2	11% (3% – 37%)	
Malignancy	5	22% (4% – 66%)	2	11% (3% – 37%)	
Cerebrovascular	0	3% (0% – 19%)	1	6% (0% – 33%)	
Unknown	5	20% (5% – 53%)	9	50% (28% – 72%)	
Other	1	3% (0% – 21%)	1	6% (0% – 33%)	

a weighted total, rounded to the nearest whole number.

**Table 4:** Graft outcomes between the continued steroid therapy (CST, reference) group and the rapid steroid withdrawal group.

	Unadjusted^a^	Model 1^b^	Model 2^c^
Graft Survival	0.79 (0.48 – 1.31)	0.81 (0.47 – 1.38)	0.71 (0.38 – 1.34)
Death-Censored Graft Survival	0.88 (0.44 – 1.77)	0.95 (0.49 – 1.85)	0.91 (0.43 – 1.91)
Patient Survival	0.69 (0.32 – 1.51)	0.62 (0.24 – 1.65)	0.57 (0.20 – 1.67)

a Unadjusted model with Inverse Probability Treatment Weight (IPTW) adjustment only;

b model 1 includes IPTW plus recipient age, gender, BMI, diabetes, pre-transplant dialysis, dialysis time, cause of ESRD (hypertension, lupus, or other vs diabetes), PRA > 30%, and Hepatitis C status;

c model 2 includes model 1 plus donor type (cadaver vs living), expanded criteria donor, donation after circulatory death, HLA matches, donor age, donor gender, donor race (black vs other), donor BMI, CMV risk (intermediate, high vs low), and transplant era (2006–2008, 2009–2012 vs 2003–2005).

**Table 5:** Post-transplant complications between the Continued Steroid Therapy (CST) and Rapid Steroid Withdrawal (RSW) Groups.

	CST (n = 150^a^)		RSW (n = 157)				

	n^a^	Weighted Mean (95% CI)	n	Weighted Mean (95% CI)	p-Value	OR (95% CI)	p-Value
GFR at 1 Year (mL/min/1.73 m^2^)	140	60.0 (51.7 – 68.3)	13 2	52.9 (50.1 – 55.8)	0.11		
Living Donor	47	51.9 (41.1 – 62.7)	52	53.4 (49.4 – 57.3)	0.80		
Deceased Donor	93	64.1 (53.5 – 74.7)	80	52.7 (48.7 – 56.6)	0.047		
GFR at 5 Years (mL/min/1.73 m^2^)	49	50.7 (40.9 – 60.6)	46	52.2 (45.6 – 58.8)	0.80		
Living Donor	18	42.9 (27.7 – 58.2)	21	53.4 (45.4 – 61.3)	0.23		
Deceased Donor	31	55.3 (44.1 – 66.5)	25	51.3 (41.1 – 61.4)	0.59		
	n^a^	Weighted% (95% CI)	n	Weighted% (95% CI)	p-Value		
Delayed Graft Function	48	32% (23% – 43%)	16	10% (6% – 16%)	<0.001	0.15 (0.06 – 0.35)	<0.001
Post-Transplant Diabetes Mellitus	28	25% (14% – 41%)	22	22% (15% – 31%)	0.66	0.85 (0.32 – 2.24)	0.74
BK Viremia	16	10% (5% – 20%)	28	18% (13% – 25%)	0.15	2.72 (1.22 – 6.04)	0.014
CMV Viremia	17	11% (5% – 21%)	37	24% (18% – 31%)	0.04	4.14 (1.81 – 9.44)	0.001
Rejection at 1 Year	15	10% (5% – 18%)	51	32% (26% – 40%)	<0.001	4.26 (1.99 – 9.13)	<0.001
Rejection at 5 Year	23	15% (9% – 25%)	69	44% (36% – 52%)	<0.001	4.71 (2.40 – 9.23)	<0.001
Biopsy Proven Rejection at 1 Year	15	10% (5% – 18%)	28	18% (13% – 25%)	0.10	2.04 (0.92 – 4.55)	0.08
Biopsy Proven Rejection at 5 Year	23	15% (9% – 25%)	37	24% (18% – 31%)	0.13	1.95 (0.98 – 3.88)	0.06
Types of Rejection (Biopsy-Proven)					<0.001		
Grade 1	5	22% (7% – 50%)	28	70% (54% – 82%)			
Grade 2	15	68% (42% – 87%)	2	5% (1% – 18%)			
Grade 3	0	0% (0% – 3%)	0	0%			
Antibody-Mediated Rejection	2	9% (3% – 24%)	5	12% (5% – 27%)			
Borderline Rejection	0	0% (0% – 2%)	5	12% (5% – 27%)			

a weighted total, rounded to the nearest whole number.

**Table 6:** Prednisone initiation in the rapid steroid withdrawal group.

	n	Percentage of RSW (n = 157)
Started on Prednisone	20	6.5%
Reasons for Starting Prednisone		
Rejection	7	35%
Leukopenia	5	25%
BK Nephropathy	2	10%
GI Intolerance	2	10%
Malignancy	1	5%
FSGS	1	5%
Pregnancy	1	5%
FK Toxicity	1	5%

## Abbreviations

AA: African-American
ACR: Acute cellular rejection
BMI: Body mass index
CMV: Cytomegalovirus
CST: Continued steroid therapy
DCD: Donation after circulatory death
ECD: Expanded criteria donors
IPTW: Inverse probability of treatment weights
PRA: panel reactive antibodies
PTDM: Post-transplant diabetes mellitus
RSW: Rapid steroid withdrawal
